# Rapid evolution in response to warming does not affect the toxicity of a pollutant: Insights from experimental evolution in heated mesocosms

**DOI:** 10.1111/eva.12772

**Published:** 2019-02-08

**Authors:** Chao Zhang, Mieke Jansen, Luc De Meester, Robby Stoks

**Affiliations:** ^1^ Evolutionary Stress Ecology and Ecotoxicology KU Leuven Leuven Belgium; ^2^ Laboratory of Aquatic Ecology, Evolution and Conservation KU Leuven Leuven Belgium

**Keywords:** ecological risk assessment, ecotoxicology, experimental thermal evolution, global warming, mesocosms, nanopollutants, thermal tolerance

## Abstract

While human‐induced stressors such as warming and pollutants may co‐occur and interact, evolutionary studies typically focus on single stressors. Rapid thermal evolution may help organisms better deal with warming, yet it remains an open question whether thermal evolution changes the toxicity of pollutants under warming. We investigated the effects of exposure to a novel pollutant (zinc oxide nanoparticles, nZnO) and 4°C warming (20°C vs. 24°C) on key life history and physiological traits of the water flea *Daphnia magna*, a keystone species in aquatic ecosystems. To address the role of thermal evolution, we compared these effects between clones from an experimental evolution trial where animals were kept for two years in outdoor mesocosms at ambient temperatures or ambient +4°C. The nZnO was more toxic at 20°C than at 24°C: only at 20°C, it caused reductions in early fecundity, intrinsic growth rate and metabolic activity. This was due to a higher accumulated zinc burden at 20°C than at 24°C, which was associated with an upregulation of a metallothionein gene at 20°C but not at 24°C. Clones from the heated mesocosms better dealt with warming than clones from the ambient mesocosms, indicating rapid thermal evolution. Notably, rapid thermal evolution did not change the toxicity of nZnO, neither at 20°C nor at 24°C, suggesting no pleiotropy or metabolic trade‐offs were at work under the current experimental design. Evaluating whether thermal evolution influences the toxicity of pollutants is important for ecological risk assessment. It provides key information to extrapolate laboratory‐derived toxicity estimates of pollutants both in space to warmer regions and in time under future global warming scenarios. In general, studying how the evolution of tolerance to one anthropogenic stressor influence tolerance to other anthropogenic stressors should get more attention in a rapidly changing world where animals increasingly face combinations of stressors.

## 1 INTRODUCTION

There is increasing appreciation that evolutionary insights are important to understand the impact of anthropogenic stressors such as warming (Stoks, Geerts, & De Meester, 2014) and pollutants (Brady, Richardson, & Kunz, 2017). This is because the strong selection induced by these stressors can lead to rapid evolutionary changes (Hendry, 2016) that may buffer their impact. Rapid evolution indeed has been shown to increase the tolerance to warming (e.g., Geerts et al., 2015; Ljungfeldt, Quintela, Besnier, Nilsen, & Glover, 2017) and to pollutants (e.g., Brady et al., 2017; Turko, Sigg, Hollender, & Spaak, 2016). While animals often are exposed to both warming and pollutants, it is largely unknown how rapid evolution imposed by one of these anthropogenic stressors shapes the ability to deal with the other one.

Two general mechanisms may underlie how the evolution of tolerance to one stressor changes the tolerance to a second stressor. Firstly, pleiotropic effects where the same set of genes influences tolerance against different stressors may generate correlated responses to selection on tolerance traits (Des Marais & Juenger, 2010). Given that genetic mechanisms of tolerance to stressors are often conserved and regulated by linked genetic networks (e.g., Rodríguez‐Verdugo, Gaut, & Tenaillon, 2013; Sikkink, Reynolds, Cresko, & Phillips, 2017) and associated with similar responses in the proteome (Sørensen, Schou, & Loeschcke, 2017), the acquisition of genetic adaptation to one stressor may increase the tolerance to another stressor (Bubliy & Loeschcke, 2005). Yet, pleiotropic effects may also be antagonistic where the evolution of increased tolerance actually reduces tolerance against a second stressor (Hua et al., 2017; Jansen, Stoks, Coors, Van Doorslaer, & De Meester, 2011). Such genetic trade‐offs have been suggested between tolerance to warming and tolerance to pollutants (“cost of tolerance concept,” Moe et al., 2013). Secondly, metabolic trade‐offs may occur where the evolution of higher levels of one function causes, through metabolic costs, lower values of another function (e.g., Arnott, Chiba, & Conover, 2006). Metabolic trade‐offs have been suggested to underlie synergistic interactions between pollutants and environmental stressors (Liess, Foit, Knillmann, Schäfer, & Liess, 2016), and to generate costs of resistance to a given stressor in terms of reduced tolerance against another stressor (Kliot & Ghanim, 2012; Pook, Lewis, & Galloway, 2009). Yet, these mechanisms are not always present and some studies documented the evolution of tolerance to one stressor without an associated change in the tolerance to a second stressor (e.g., Bono, Smith, Pfennig, & Burch, 2017).

The potential of rapid thermal evolution shaping the toxicity of pollutants in a warming world is highly relevant for ecological risk assessment. There is growing concern that current risk assessment does not accurately assess the impact of pollutants in a warming world (Landis et al., 2013; Noyes & Lema, 2015). Moreover, standard laboratory ecotoxicology tests are typically done at ambient temperatures, but the results are extrapolated to entire species’ ranges where higher temperatures may prevail in some regions. Many pollutants, such as many metals and organophosphate pesticides, are more toxic at higher temperatures (e.g., Debecker, Dinh, & Stoks, 2017; Holmstrup et al., 2010; Noyes et al., 2009). Yet, this pattern is not general (e.g., Perschbacher, 2005; Scheil & Köhler, 2009; Talent, 2005), and for many pollutant groups, we still lack information on the temperature dependency of their toxicity. The latter include nanoparticles, a group of “pollutants of emerging concern” that is getting increasing attention due to their environmental release at a large scale and their ecotoxicity (Bour, Mouchet, Silvestre, Gauthier, & Pinelli, 2015; Selck, Handy, Fernandes, Klaine, & Petersen, 2016). To arrive at a realistic assessment of the impact of nanoparticles in a warming world, we need to understand how their toxicity is shaped by temperature and how this is further modulated by rapid thermal evolution.

Nano zinc oxide (nZnO) particles are among the most produced nanoparticles worldwide with an annual global production of about 550 tons (Piccinno, Gottschalk, Seeger, & Nowack, 2012). Nano ZnO is widely used in plastics, glass, food, batteries and personal care products such as sunscreens. Accordingly, nZnO particles have been detected in the environment at concentrations of up to 17.1 and 23.2 mg per kg dry weight in sludge in Europe and the United States, respectively (Ma, Williams, & Diamond, 2013; Read et al., 2016). The physiological toxic mode of action of nZnO includes the generation of reactive oxygen species (ROS) that leads to oxidative damage, the inhibition of several antioxidant enzymes (e.g., SOD, CAT and GPx) and the disruption of cellular zinc homeostasis which leads to mitochondria damage and ultimately cell death (Ma et al., 2013; Xia et al., 2008).

Here, we studied the single and combined effects of the novel pollutant nZnO and warming on the life history and physiology of the water flea *Daphnia magna* and whether these effects were modulated by rapid thermal evolution using an experimental evolution approach. The water flea *D. magna* is a keystone species in aquatic ecosystems (Miner, De Meester, Pfrender, Lampert, & Hairston, 2012) and a model species in evolutionary ecotoxicology (Jansen, Coors, Stoks, & De Meester, 2011). Recently, we have shown using resurrection ecology that a natural *D. magna* population evolved a higher tolerance to 4°C warming across a 40‐year period and that this thermal evolution could offset the change in toxicity of nZnO under warming (Zhang, Jansen, De Meester, & Stoks, 2018). In the same population, an evolutionary increase in tolerance of extreme temperatures (measured as the critical thermal maximum, CTmax) has also been documented (Geerts et al., 2015). Resurrection ecology is a “back‐in‐time” approach where past, realized evolution that occurred in a natural population can be studied by comparing recent individuals with the resurrected ancestors (Franks, Hamann, & Weis, 2018). Given its power and high level of realism, it has been commonly used (e.g., Goitom et al., 2018; Lenormand et al., 2018; Stoks, Govaert, Pauwels, Jansen, & De Meester, 2016). Yet, an important limitation for resurrection ecology is that it cannot unambiguously reveal the specific causes for the evolutionary shifts as many factors may have changed through time. Instead, we used here experimental evolution, a “forward‐in‐time” approach to assess evolutionary change (Franks et al., 2018) where experimental populations are exposed to well‐known, controlled selection pressures and tested after a certain number of generations (Kawecki et al., 2012). This allows more direct coupling of the specific selective agent and the observed evolutionary change. Moreover, it easily allows replicated evolutionary trials, while resurrection ecology is often limited to a single population that is followed through time (a single natural population was studied in Zhang et al., 2018). Another important difference is that while in the Zhang et al. (2018) study more gradual evolution occurred during a long time period (40 years), in the current study we tested for rapid thermal evolution during a 2‐year period.

We here capitalized on a thermal selection experiment where *D. magna* clones were kept in outdoor mesocosms under ambient or ambient +4°C temperatures (Feuchtmayr et al., 2009). Clones from the heated mesocosms evolved a higher tolerance to deal with extremely high temperatures as measured by CT_Max_ (Geerts et al., 2015). Given the potential of *D. magna* populations to rapidly evolve in response to higher temperatures both in terms of tolerance to mild 4°C warming (e.g., Zhang, Jansen, De Meester, & Stoks, 2016; Zhang et al., 2018) and in terms of extreme heat tolerance (e.g., Brans et al., 2017; Geerts et al., 2015), we expected the clones from the heated mesocosms to perform also better under mild 4°C warming compared to the clones from the ambient mesocosms. Given the widespread occurrence of interactions between warming and heavy metals (Van Dinh et al., 2013; Sokolova & Lannig, 2008), we expected the effects of nZnO to depend on temperature and this dependence to be smaller under thermal evolution. To explore the mechanisms underlying nZnO effects, we determined internal body zinc burdens and the gene expression of metallothioneins which are key metal detoxification proteins (Amiard, Amiard‐Triquet, Barka, Pellerin, & Rainbow, 2006; Shaw et al., 2007).

## 2 MATERIALS AND METHODS

### 2.1 Outdoor selection experiment and study animals

A two‐year thermal selection experiment simulating global warming in outdoor mesocosms under semi‐natural conditions was conducted between October 2005 and September 2007 at Ness Botanic Gardens, a nature area unlikely to have been exposed to metal pollution, in north‐western England (53°16′N, 3°03′W). Detailed information on the mesocosm experiment is given in Feuchtmayr et al. (2009). Briefly, each mesocosm contained 3,000 L groundwater and a 20 cm deep sediment mixture from an uncontaminated source pond that did not contain any *D. magna* resting eggs. At the start of the experiment, *D. magna* resting eggs from a nearby shallow pond were thoroughly mixed and then inoculated in each mesocosm. The *Daphnia* clones were never exposed to nZnO or Zn ions during the experimental evolution trials. There were two thermal selection regimes: mesocosms were exposed to either ambient temperatures (unheated) or to ambient +4°C (heated) thereby simulating 4°C warming by 2100 as predicted by IPCC scenario RCP8.5 (IPCC, 2013). After two years of selection, sediment was collected from each mesocosm. We only used the top 2 cm sediment to ensure that any resting eggs present were recent, hence formed at the end of the two‐year selection experiment. Note that any accidental inclusion of older eggs would make it harder to detect a signal of thermal evolution. Sediment with resting eggs was stored at 4°C in the dark until *D. magna* clones were hatched from the resting eggs in these sediments in 2009. Thereafter, the hatchlings were used to establish clonal lineages and kept in monoclonal culture under standard laboratory conditions (20°C, photoperiod of 14:10 L:D, fed 1×10^5^ cells/ml green alga *Scenedesmus obliquus* twice a week). As dormant eggs are produced sexually in *D. magna*, all hatchlings are genetically unique.

We started the exposure experiment in November 2015 with seven randomly chosen clones from two ambient mesocosms and seven randomly chosen clones from two heated mesocosms. This resulted in a total of 14 clone lineages. Those clones were a subset from the study by Geerts et al. (2015). To minimize interference from maternal effects, all animals were cultured for several generations (ca. three months) under standard experimental conditions (20°C, photoperiod of 14:10 L:D, fed 1×10^5^ cells/ml *S. obliquus* daily, medium refreshed every other day). This was done in ISO 6341 medium (CaCl_2_•2H_2_O: 0.294 g/L, MgSO_4_•H_2_O: 0.123 g/L, NaHCO_3_: 0.065 g/L, KCl: 0.006 g/L) which is recommended for toxicity testing of metals (OECD, 2004).

### 2.2 Nanopollutant exposure

A nano zinc oxide dispersion (20 wt.% in H_2_O) was purchased from Sigma (Sigma‐Aldrich, Louis, MO, USA). The nanoparticles were mostly spherical with an average size less than 50 nm (characterized by a transmission electron microscope, Zeiss EM900, Carl Zeiss, Oberkochen, Germany). A stock solution of 5 × 10^3^ mg/L Zn was prepared by sonication in an ultrasonic bath (Elmasonic S40, Elma^®^, Germany) for 30 min and stored at 4°C under darkness. Each time, a test solution was made, and the stock solution was ultrasonicated for 30 min at 20 kHz with a maximum power output of 400 W to eliminate aggregates. A sublethal exposure concentration of nZnO (86 µg/L Zn) was used. This concentration corresponds to 10% of the EC_50, 48 hr_ immobilization for *D. magna* neonates based on a pilot range‐finding experiment. The EC_50, 48 hr_ immobilization is defined as the concentration at which half of the *Daphnia* individuals were not moving anymore after 48 hr of exposure. This concentration is similar to chronic EC_50_ values of nZnO for *D. magna* reproduction (Adam et al., 2014) and is environmentally relevant as the estimated nZnO concentrations in UK waterbodies go up to 100 μg/L (Boxall, Tiede, & Chaudhry, 2007).

### 2.3 Experimental set‐up

To investigate the effects of nZnO and test temperature on key life history and physiological traits of clones that did and did not undergo thermal selection, a full factorial experiment was set up where clones of each thermal selection regime (ambient vs. heated mesocosms) were exposed to two nZnO treatments (nZnO absent vs. present) and two test temperatures (20°C vs. 24°C). The test temperatures correspond to the mean summer temperature in the ambient mesocosms (20°C) and in the heated mesocosms (24°C) (Van Doorslaer et al., 2010). Because we focus on the different responses between the two thermal selection regimes, the seven clones per selection regime were used as replicates.

The mothers of the experimental *Daphnia* were reared in 500‐ml glass vials filled with 450 ml ISO 6341 medium at 20°C and 24°C for one generation until they released their second clutch. Neonates from the second clutch were randomly divided across the two nZnO treatments and kept at the same temperature treatment as their mothers. For each of the eight treatment combinations, we exposed a set of 15–17 individuals of each clone in a 500‐ml vial. In total, there were ca 850 individuals for the whole experiment that was conducted in one time block. *Daphnia* were fed daily with *S. obliquus* (1×10^5^ cells/ml), and the culture medium was refreshed every other day. Juveniles were daily counted and removed from the vials. The animals in the cohorts developed under each treatment combination sufficiently synchronously so that we could stop vials when all animals had released their second clutch and while there were no visual signs of the third clutch in the brood pouches. Afterwards, for each treatment combination, *Daphnia* were flash‐frozen in three separate Eppendorf tubes with liquid nitrogen and stored at −80°C for later analyses.

Directly after the renewal of the medium, the zinc concentrations were measured using ICP‐MS (inductively coupled plasma mass spectrometry, Agilent 7700x, Biocompare, USA) after samples were acidified with HNO_3_. The zinc concentrations were 72.01 ± 2.14 µg/L at 20°C and 73.24 ± 2.45 µg/L at 24°C (mean ± *SD*,* n* = 5 pooled vials). The associated water quality parameters were as follows: pH: 7.99 ± 0.03, conductivity: 623.23 ± 1.55 µS/cm, dissolved oxygen: 8.99 ± 0.19 mg/L and hardness: 239.10 ± 2.75 mg/L CaCO_3_. These values are within the range encountered in natural *D. magna* populations (e.g., Orsini, Spanier, & De Meester, 2012).

### 2.4 Response variables

Three key life history variables of *Daphnia* were quantified as follows: age at first reproduction (the age at which the first juveniles were seen in a jar), early fecundity (total number of juveniles of the first two broods) and intrinsic growth rate. The latter was calculated based on the timing and the size of the first two clutches using the Euler equation (Roff, 2001): $1=\int e^{-rx}l_{x}m_{x}dx$. Here, *l_x_* represents the proportion of survivors at age *x*, and *m_x,_* the number of offspring released at day *x*. All variables were recalculated to values per jar.

One set of ten pooled *Daphnia* from a given vial was used to measure the internal Zn content. *Daphnia* were first homogenized in 150 μl Milli‐Q water to generate ca. 200 μl homogenate. Forty microlitres of the homogenate was dried at 60°C for 24 hr in preweighed tin capsules and used to measure the dry body mass. Another 120 μl aliquot of the homogenate was used for measuring total Zn (including the body Zn; Zn is an essential trace metal) contents after digestion with HNO_3_ using inductively coupled plasma mass spectrometry (Agilent 77009 ICP‐MS; Biocompare, South San Francisco, CA, USA). The internal Zn content was expressed as µg per mg dry mass.

The RNA:DNA ratio was measured following Vrede, Persson, and Aronsen (2002) using one *Daphnia* per vial. The RNA:DNA ratio is considered a good proxy for metabolic activity because the total RNA content is primarily a function of the ribosome number, whereas the DNA content remains constant in an individual (Pauwels, Stoks, & De Meester, 2010). Animals were first homogenized in extraction buffer (50 mM EDTA, 0.05% SDS in 50 mM Tris), and then, a mixture of 100 µl homogenate and 2 µl ethidium bromide (100 µg/ml) was incubated on ice for 15 min. The total amount of RNA + DNA was measured at an excitation/emission wavelength of 535:595 nm. Next, the RNA was broken down by adding 1 µl of RNase solution (20 mg/ml) to another 100 µl homogenate and incubating at room temperature for one hour. Afterwards, the remaining DNA was measured. RNA and DNA concentrations were measured in triplicate, and the means per sample were used for the statistical analyses.

The gene expression of two metallothionein genes (MTa, MTb) that are involved in protection against metals was measured on four pooled *Daphnia* per vial. First, total RNA was extracted using TRIzol (Invitrogen, Belgium) with DNase treatment. The purity and concentration of RNA were measured with a NanoDrop ND‐1000 spectrophotometer (NanoDrop Technologies). Then, 300 ng of extracted RNA was reverse‐transcribed to cDNA using the QuantiTect Reverse Transcription Kit (Qiagen). 18S rRNA was assayed to normalize for total cDNA in each sample. Primer sequences and the qRT‐PCR protocol were based on Poynton et al. (2008). A SYBR Green Master Mix was used for the qRT‐PCR with an ABI Prism 7000 Sequence Detection System (Applied Biosystems, Foster City, CA, USA) under the following conditions: 2 min at 95°C and 40 cycles each consisting of 15 s at 95°C and 1 min at 60°C. The melting curve was included to verify amplification specificity. RT‐PCR data were analysed using GenEx software (version6, MultiD) for quality control, normalization, transformation and gene expression analysis.

### 2.5 Statistical analyses

Effects of nZnO exposure (Zn), test temperature in the laboratory (TTemp) and selection temperature in the outdoor mesocosms (STemp) on life history and physiological traits were analysed with separate linear mixed models (LMMs) with a normal error distribution using the packages lme4 (Bates, Maechler, Bolker, & Walker, 2014), car (Fox & Weisberg, 2011), effects (Fox, 2003) and lsmeans (Lenth, 2016) in R version 3.4.1 (R Core Team, 2013). In each model, all interactions between nZnO exposure, test temperature and selection temperature (as fixed factors) were included. In addition, we added clone nested in mesocosm and mesocosm nested in selection temperature as random factors; their effects were tested using the rand function. The model equation in R for each trait is as follows: model = lmer (Trait_x_ ~ Zn * TTemp * STemp + (1|Mesocosms/Clones), Data). The significance of the explanatory variables was determined using Wald chi‐square tests. Following Moran (2003), significance levels were not corrected for multiple testing. Total zinc content was log‐transformed. *P* values <0.05 were considered significant.

## 3 RESULTS

### 3.1 Internal Zn concentrations

**Figure 1:**
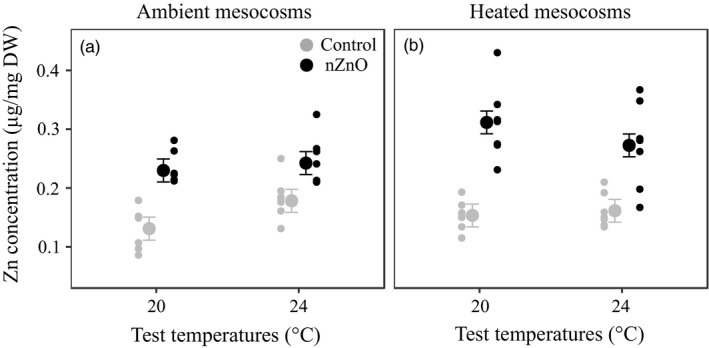
Body total zinc concentrations as a function of nZnO and test temperature in the clones from ambient (a) and heated mesocosms (b). Given are least‐squares means ±1 *SE* and the raw data of the seven clones

No mortality occurred during the experiment. Exposure to nZnO increased body Zn burdens, and the percentage of increase relative to the nonexposed control at 20°C was almost two times higher at 20°C (92.7%) than at 24°C (50.9%) (main effect of Zn and Zn × TTemp interaction, Table 1, Figure 1). The zinc concentration in the clones from the heated mesocosms was higher than in the clones from the ambient mesocosms at 20°C (TTemp × STemp, Table 1, Figure 1).

**Table 1:** Results of the linear mixed models testing for the effects of nZnO (Zn), test temperature (TTemp) and selection temperature (STemp) on life history and physiological traits of *Daphnia magna*

Variables	Zn		TTemp		STemp		Zn × TTemp		TTemp × STemp		Zn × STemp		Zn × TTemp × STemp	
	$\chi_{1}^{2}$	*p*	$\chi_{1}^{2}$	*p*	$\chi_{1}^{2}$	*p*	$\chi_{1}^{2}$	*p*	$\chi_{1}^{2}$	*p*	$\chi_{1}^{2}$	*p*	$\chi_{1}^{2}$	*p*
Life history														
Intrinsic growth rate	31.29	**<0.001**	11.08	**<0.001**	17.61	**<0.001**	13.25	**<0.001**	45.43	**<0.001**	0.78	0.378	0.99	0.319
Age at 1st reproduction	12.19	**<0.001**	22.19	**<0.001**	4.76	**0.029**	0.76	0.383	9.33	**0.002**	0.19	0.663	0.19	0.663
Early fecundity	20.09	**<0.001**	205.56	**<0.001**	3.44	0.063	16.02	**<0.001**	8.43	**0.003**	0.19	0.663	0.87	0.351
Physiology														
Zn burdens	92.99	**<0.001**	1.20	0.273	1.59	0.208	5.11	**0.024**	5.02	**0.025**	2.46	0.116	0.05	0.817
RNA:DNA	46.20	**<0.001**	33.97	**<0.001**	0.60	0.425	7.93	**0.005**	12.23	**<0.001**	0.95	0.329	0.19	0.665
MTa	3.59	0.058	0.81	0.370	0.49	0.483	0.00	0.996	1.98	0.159	1.66	0.198	0.42	0.516
MTb	18.21	**<0.001**	3.46	0.063	1.40	0.236	6.57	**0.010**	0.47	0.495	2.50	0.114	0.41	0.522

Note

Significant *p*‐values are marked in bold.

### 3.2 Life history

**Figure 2:**
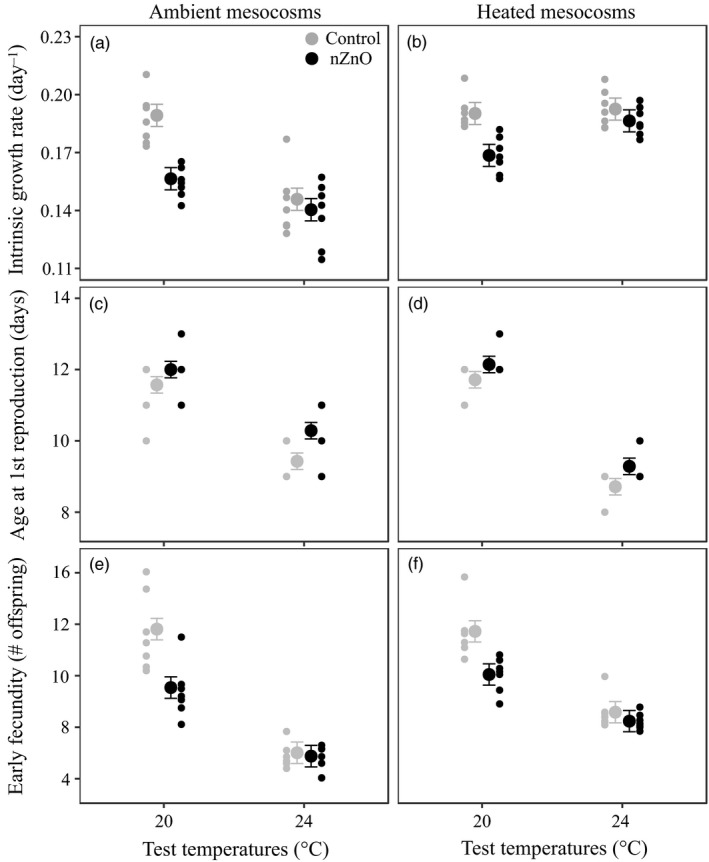
Intrinsic growth rate, age at 1st reproduction and early fecundity of *Daphnia magna* as a function of nZnO and test temperature in the clones from ambient (a, c, e) and heated mesocosms (b, d, f). Given are least‐squares means ±1 *SE* and the raw data of the seven clones

Exposure to nZnO decreased *Daphnia* intrinsic growth rate when tested at 20°C but not at 24°C (Zn × TTemp, Table 1, Figure 2a,b). This was because under nZnO exposure, *Daphnia* reproduced later at both temperatures (main effect of Zn, Table 1, Figure 2c,d), and only had a lower early fecundity at 20°C (Tukey *p* < 0.001), but not at 24°C (Tukey *p = *0.86) (Zn × TTemp, Table 1, Figure 2e,f). The clones from the ambient mesocosms showed a less strong acceleration of development at 24°C and suffered a stronger reduction in early fecundity at 24°C compared to the clones from the heated mesocosms; as a result, only the clones from ambient mesocosms showed a reduction in intrinsic growth rate at 24°C (Tukey, ambient mesocosms: *p* < 0.001; heated mesocosms: *p* = 0.09) (TTemp × STemp, Table 1, Figure 2). This translated in clones from the heated mesocosms having a younger age at first reproduction, a higher early fecundity and intrinsic growth rate than the clones from the ambient mesocosms when tested at 24°C (Tukey all *p* < 0.05), while no life history differences between clones from the two thermal selection treatments were apparent at 20°C (Tukey all *p* > 0.79) (TTemp × STemp, Table 1, Figure 2). This evolution of a higher thermal tolerance in the clones from the heated mesocosms did not change the general tolerance to nZnO (no significant Zn × STemp interactions) neither the tolerance to nZnO under higher test temperatures (no significant Zn × TTemp × STemp interactions, Table 1). Note, the effect sizes of our key fitness measure (intrinsic growth rate) for the two relevant interaction terms were low (eta‐squared values, Zn × STemp: 0.016, Zn × TTemp × STemp: 0.021), indicating, if anything biological significance of these interactions to be low.

### 3.3 Physiology

**Figure 3:**
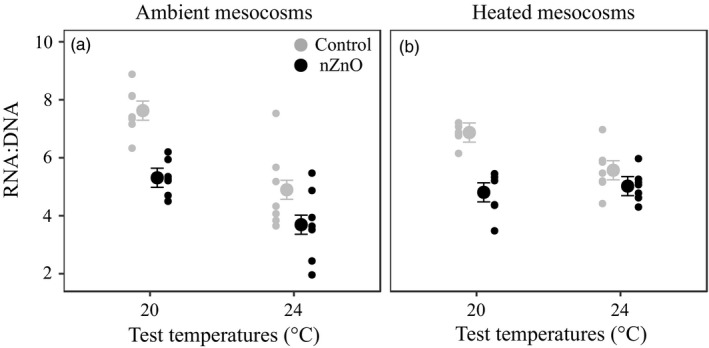
RNA:DNA ratio of *Daphnia magna* as a function of nZnO and test temperature in the clones from ambient (a) and heated mesocosms (b). Given are least‐squares means ±1 *SE* and the raw data of the seven clones

Exposure to nZnO reduced the RNA:DNA ratio, and this was stronger at 20°C (−30.2%) than at 24°C (−16.8%) (main effect Zn and Zn × TTemp, Table 1, Figure 3a,b). At the high test temperature, the RNA:DNA ratio was lower in the clones from the ambient mesocosms but not in the clones from the heated mesocosms (main effect TTemp and TTemp × STemp, Table 1, Figure 3a,b). The DNA content did not differ between the clones from the ambient and heated mesocosms (main effect STemp: $\chi_{1}^{2}$ = 0.88, *p* = 0.35).

**Figure 4:**
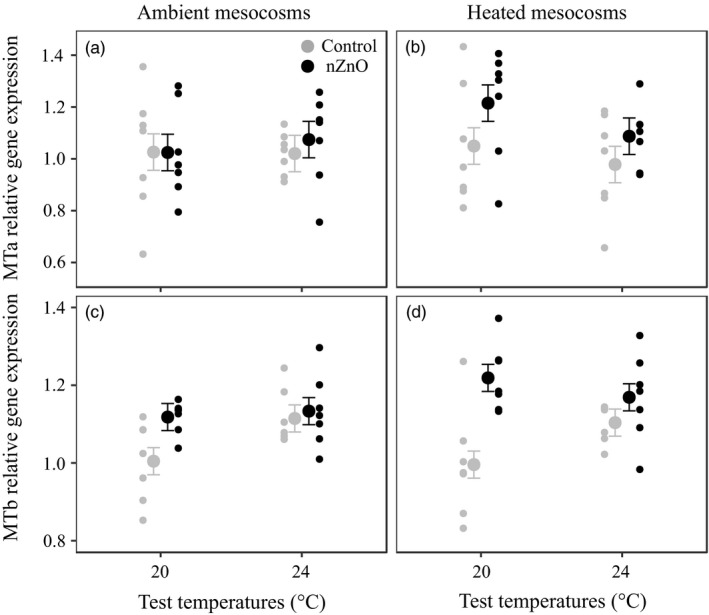
Relative expressions of MTa and MTb of *Daphnia magna* as a function of nZnO and test temperature in the clones from ambient (a, c) and heated mesocosms (b, d). Given are least‐squares means ±1 *SE* and the raw data of the seven clones

There was a trend for an upregulation of the MTa gene expression due to nZnO exposure (*p* = 0.058, Table 1, Figure 4a,b). Exposure to nZnO increased the MTb gene expression at 20°C but not at 24°C (Table 1, Figure 4c,d).

## 4 DISCUSSION

As expected, exposure to the sublethal concentration of nZnO negatively affected life history and metabolic rate of this keystone aquatic species. While the toxicity of nZnO was dependent on temperature, surprisingly nZnO‐induced effects were stronger at 20°C compared to 24°C. Hence, both stressors (warming and nZnO) did not magnify each other's effects, instead metal toxicity declined at higher temperatures. Using an experimental evolution approach, we found a strong signal of thermal evolution with clones from the heated mesocosms dealing better with warming. This rapid thermal evolution, however, had no overall effect on the sensitivity to nZnO neither at 20°C nor at 24°C. In other words, and in contrast with the cost of tolerance concept (Moe et al., 2013), the evolution of increased tolerance to heat stress was not traded off against a reduced tolerance to nZnO.

### 4.1 Temperature‐dependent effects of nZnO on life history and physiology

The negative effects of nZnO exposure at 20°C on life history (delayed reproduction, reduced early fecundity and decreased intrinsic growth rate) and decreased metabolic rate (as measured by the RNA:DNA ratio) are in accordance with previous results (Adam et al., 2014) and responses to other metal oxide nanoparticles (Zhu, Chang, & Chen, 2010) in the study species. Notably, despite the general pattern of a stronger toxicity of metals at higher temperatures (Debecker et al., 2017; Holmstrup et al., 2010; Noyes et al., 2009), the negative effects of nZnO on early fecundity and intrinsic growth rate were only present at 20°C and not at 24°C. This could be explained by the stronger increase in the zinc burden at 20°C than at 24°C. The here observed pattern of a greater toxicity of metals at lower temperature has been observed before, for example, in channel catfish *Ictalurus punctatus* exposed to copper where it was presumably driven by the reduced functioning of defence mechanisms at lower temperatures (Perschbacher, 2005). This explanation seems less likely in our study as the metallothionein gene MTb was instead upregulated at 20°C and not at 24°C. As metallothioneins are key proteins involved in metal detoxification (Amiard et al., 2006), the higher toxicity and MT upregulation at 20°C also matched the stronger increase in zinc burden at 20°C than at 24°C. An increased accumulation of zinc in *Daphnia* has been causally linked to a reduced early fecundity (Adam et al., 2014). The smaller uptake of nZnO at 24°C was likely caused by a lower feeding rate of the *Daphnia* under this stressful temperature. The uptake of Zn is indeed associated with feeding on algae in *Daphnia* (Memmert, 1987), and grazing rates typically decrease under stressful conditions (Lari, Gauthier, Mohaddes, & Pyle, 2017).

In contrast, in a previous study, we showed the here used concentration of nZnO to be more toxic under 4°C warming for *D. magna* (Zhang et al., 2018). One possible reason for this difference with current study is that the type of interaction between stressors may strongly depend on the severity of the individual stressors (Côté, Darling, & Brown, 2016; Kaunisto, Ferguson, & Sinclair, 2016). Although we used the same temperatures and nZnO concentration in both studies, differences in nZnO sensitivity of the clones used in both studies may have contributed to different interaction patterns. Indeed, when exposed to nZnO at 20ºC, the intrinsic growth rate in the current study was generally lower than in the previous study, indicating the same concentration of nZnO was more toxic to the *Daphnia* clones in the current study. The only other study that tested the toxicity of a nanopollutant under a realistic warming scenario found that 4°C warming magnified the deleterious effect of nZnO on fertilization in the sea urchin *Tripneustes gratilla* possibly because the 4°C warming was near the upper thermal tolerance (Mos, Kaposi, Rose, Kelaher, & Dworjanyn, 2017). But this effect was only found at a very high concentration of nZnO (10 mg/L). At lower concentrations (including the here used concentration), 4°C warming had no impact on the effects of nZnO. This confirms that the effect of temperature on the toxicity of nanopollutants may be concentration dependent (Mos et al., 2017). Together with our result, this underlines the difficulty in predicting the effects of nanopollutants under warming.

### 4.2 Rapid thermal evolution and the sensitivity to nZnO

Capitalizing on a two‐year thermal selection experiment, our results indicate rapid thermal evolution of *D. magna* clones from the heated mesocosms. Indeed, the warming‐induced negative effects on early fecundity, intrinsic growth rate and metabolic activity as seen in the clones from the ambient mesocosms were less strong or even absent in the clones from the heated mesocosms. The here observed evolution of a higher tolerance to chronic mild 4°C warming complements the evolution of a higher acute tolerance to deal with heat extremes in the clones from the heated mesocosms (Geerts et al., 2015). Thermal evolution allows the heat‐adapted genotypes to better deal with warming, hence might reduce the impact of the predicted warming by the end of this century.

There are several studies demonstrating thermal evolution under global warming conditions in aquatic animals (Stoks et al., 2014; for the studied species: Geerts et al., 2015; Van Doorslaer et al., 2009) and temperature‐dependent effects of pollutants (e.g., Debecker et al., 2017; Morin et al., 2017). We integrated both patterns in a single study which allowed us to demonstrate that rapid experimental thermal evolution unexpectedly did not have an overall effect on the temperature‐dependent sensitivity to the here studied pollutant. Noteworthy, a recent study by Freitas et al. (2017) showed plastic nongenetic adaptation to warming (pre‐exposure to warming conditions for 14 days) also did not increase tolerance to the trace metal Hg in mussels under warming.

Evolution of tolerance to one stressor may cause changes in the tolerance to a second stressor in case of pleiotropic effects (e.g., Des Marais, Hernandez, & Juenger, 2013) and metabolic trade‐offs (e.g., Kliot & Ghanim, 2012; Pook et al., 2009). Hence, our results suggest no pleiotropic effects between tolerance to warming and tolerance to this pollutant despite stress response networks being generally evolutionary conserved, hence many genes shared by different stress response pathways (e.g., Gasch et al., 2000). This may suggest that the typical stress response pathways to warming and to the here studied nanopollutant showed less overlap. Yet, even when such overlap is expected to be high, correlated responses to selection imposed by one stressor are not always observed and may even switch sign depending on the environment (Sikkink et al., 2017). Furthermore, our results suggest that the evolution of thermal tolerance was not associated with a high metabolic cost (Clarke, 2003) that caused less investment in defence against this pollutant. Our results match other studies that did not document evolutionary trade‐offs when dealing with different stressors (e.g., Bono et al., 2017).

Our current findings based on experimental evolution contrast with those of a resurrection study where thermal evolution across ca. 40 years did change the toxicity of the same concentration of nZnO in *D. magna* (Zhang et al., 2018). The reasons for this contrasting result may be related to the fundamentally different way how thermal evolution was studied. In current experimental evolution study, *Daphnia* was exposed to an “abrupt” (ambient +4°C) warming scenario for a short period (two years), while in the resurrection ecology *Daphnia* was exposed to a more gradual and more subtle mean temperature increase (ca. 1.2°C, Met Office, 2012), yet for a much longer time (ca. 40 years). In addition, in the resurrection study, there were multiple heat waves throughout the years (Met Office, 2012) to which *Daphnia* has been shown to evolve thermal tolerance (Zhang et al., 2016). While both experimental approaches resulted in the evolution of a higher ability to deal with extreme temperatures (measured as CTmax), this evolutionary response was stronger in the experimental evolution trial (Geerts et al., 2015). Given a widespread trade‐off between tolerance of a short‐term acute thermal stress and long‐term mild thermal stress (Rezende, Castañeda, & Santos, 2014), possibly *Daphnia* from the experimental evolution trials evolved a less strong increase in tolerance to deal with 4°C mild warming. Indeed, the evolutionary increase in the intrinsic growth rate (a key fitness trait) under warming in the current experimental evolution study (1.2%) was less strong than that in the resurrection study (6.3%). More general, the ecological contexts were also different between these two studies, while in the current experimental evolution study an artificial zooplankton community was established, in the resurrection study *Daphnia* encountered a fully natural lake community including predators and other competitors. The ecological context may generate changes in the selection pressure and the resulting evolutionary responses to thermal selection (De Meester, Van Doorslaer, Geerts, Orsini, & Stoks, 2011) and may change and even reverse the sign of pleiotropic effects between the tolerances against different stressors (Sikkink et al., 2017).

### 4.3 Conclusions and implications

It remains challenging to predict and understand the impact of pollutants under warming (Moe et al., 2013; Noyes et al., 2009). By studying the effects of a worldwide nanopollutant under a realistic global warming scenario and the potential interference from experimental thermal evolution, our study added two novel insights at the intersection of global change biology and evolutionary ecotoxicology that are important to address the challenge of improving risk assessment in a warming world (Landis et al., 2013). First, the here studied nanopollutant did not follow the general pattern of a higher toxicity at higher temperatures (Debecker et al., 2017; Holmstrup et al., 2010; Noyes et al., 2009). Temperature‐dependent effects of nanopollutants should receive more attention because their effects under global warming may be complex and hence difficult to predict (Mos et al., 2017). Second, and more importantly, rapid experimental thermal evolution did not change the effects of this nanopollutant under warming. This is consistent with the finding by Op de Beeck, Verheyen, and Stoks (2017) of no changed tolerance to a pesticide under gradual thermal evolution linked to a latitudinal gradient, but contrasts with a previous study (Zhang et al., 2018) suggesting that the selection regime and ecological context may critically shape the evolutionary outcome of multi‐stressor interactions. More studies (including more concentrations of pollutants, temperatures, and species) are needed to identify the conditions when genetic adaptation to warming changes the effects of pollutants under warming. We have in general poor knowledge how genetic adaptation to one anthropogenic stressor influences its interactions with other anthropogenic stressors (but see e.g., Hua et al., 2017; Jansen, Stoks et al., 2011; Sikkink et al., 2017). Yet, such multi‐stressor evolutionary studies will be of crucial importance to improve risk assessment in a rapidly changing world where animals increasingly face combinations of stressors.

## CONFLICT OF INTEREST

None declared.

## DATA ARCHIVING STATEMENT

Data for this study are available from the Dryad Digital Repository: https://doi.org/10.5061/dryad.rm5c4qr
